# Supplementary material for: Netcombin: An algorithm for constructing optimal phylogenetic network from rooted triplets
Source: PLoS One. 2020 Sep 18;15(9):e0227842. doi: 10.1371/journal.pone.0227842 (PMC7500971; doi:10.1371/journal.pone.0227842)
Supplement: S4 File — (ZIP) [file pone.0227842.s004.zip › SequenceData-Triplet/sequenceData-10-20-30-0-Triplet/length 300, numseq 40, rate 10^-7/7 seq.pdf]

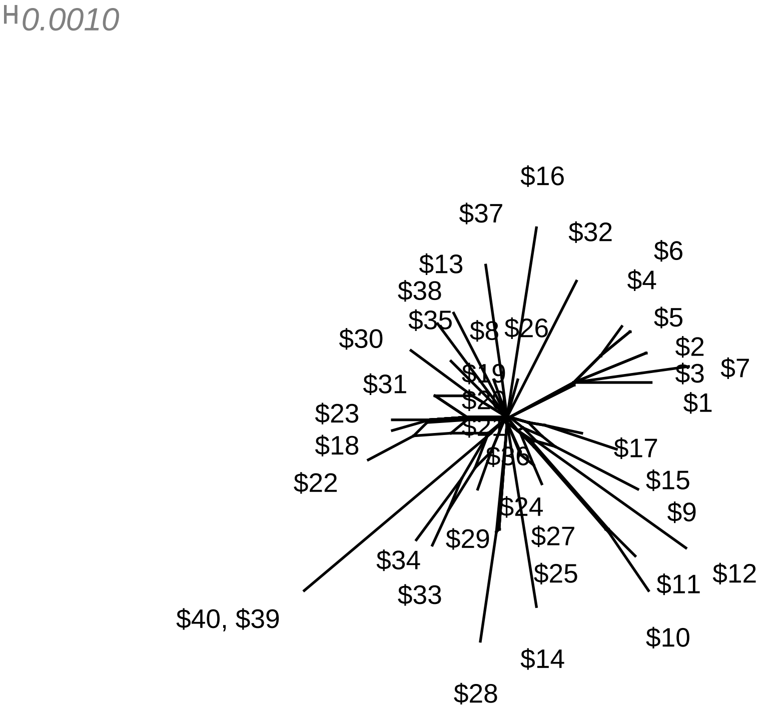
0.0010
$16
$37
$32
$6
$13
$4
$38
$5
$35
$8
$26
$30
$2
$7
$19
$3
$31
$1
$23
$18
$17
$22
$36
$15
$9
$24
$27
$29
$34
$12
$25
$11
$33
$40, $39
$10
$14
$28
